# Multiple processes independently predict motor learning

**DOI:** 10.1186/s12984-020-00766-3

**Published:** 2020-11-17

**Authors:** Christopher M. Perry, Tarkeshwar Singh, Kayla G. Springer, Adam T. Harrison, Alexander C. McLain, Troy M. Herter

**Affiliations:** 1 Department of Exercise Science, University of South Carolina, Columbia, SC 29208 USA; 2 Department of Kinesiology, The Pennsylvania State University, University Park, PA 16802 USA; 3 Department of Epidemiology and Biostatistics, University of South Carolina, Columbia, SC 29208 USA

**Keywords:** Motor learning, Motor control, Visual search, Eye-hand coordination, Visuomotor

## Abstract

**Background:**

Our ability to acquire, refine and adapt skilled limb movements is a hallmark of human motor learning that allows us to successfully perform many daily activities. The capacity to acquire, refine and adapt other features of motor performance, such as visual search, eye-hand coordination and visuomotor decisions, may also contribute to motor learning. However, the extent to which refinements of multiple behavioral features and their underlying neural processes independently contribute to motor learning remains unknown. In the current study, we used an ethological approach to test the hypothesis that practice-related refinements of multiple behavioral features would be independently predictive of motor learning.

**Methods:**

Eighteen healthy, young adults used an upper-limb robot with eye-tracking to practice six trials of a continuous, visuomotor task once a week for six consecutive weeks. Participants used virtual paddles to hit away 200 “Targets” and avoid hitting 100 “Distractors” that continuously moved towards them from the back of the workspace. Motor learning was inferred from trial-by-trial acquisition and week-by-week retention of improvements on two measures of task performance related to motor execution and motor inhibition. Adaptations involving underlying neural processes were inferred from trial-by-trial acquisition and week-by-week retention of refinements on measures of skilled limb movement, visual search, eye-hand coordination and visuomotor decisions. We tested our hypothesis by quantifying the extent to which refinements on measures of multiple behavioral features (predictors) were independently predictive of improvements on our two measures of task performance (outcomes) after removing all shared variance between predictors.

**Results:**

We found that refinements on measures of skilled limb movement, visual search and eye-hand coordination were independently predictive of improvements on our measure of task performance related to motor execution. In contrast, only refinements of eye-hand coordination were independently predictive of improvements on our measure of task performance related to motor inhibition.

**Conclusion:**

Our results provide indirect evidence that refinements involving multiple, neural processes may independently contribute to motor learning, and distinct neural processes may underlie improvements in task performance related to motor execution and motor inhibition. This also suggests that refinements involving multiple, neural processes may contribute to motor recovery after stroke, and rehabilitation interventions should be designed to produce refinements of all behavioral features that may contribute to motor recovery.

## Introduction

Humans learn to perform a broad repertoire of motor tasks that often require diverse and adaptable limb movements (i.e., skilled limb movements) to interact with our outside world. Many motor tasks, such as cooking, walking and driving, also employ diverse and adaptable patterns of eye movements (i.e., visual search) to actively gather visual information for planning and execution of skilled limb movements. Information gathered by visual search is also used to decide what skilled limb movements should be performed to achieve task goals (i.e., visuomotor decisions). Conversely, patterns of visual search are influenced by the available repertoire of skilled limb movements that can be used to achieve task goals. These interactions between skilled limb movements and visual search lead to coordinated patterns of eye and limb movements (e.g., eye-hand coordination). Overall, skilled limb movements, visual search, eye-hand coordination and visuomotor decisions may all contribute to learning and performance of motor tasks. However, we do not know the extent to which these behavioral features and their underlying neural processes are independently refined to produce improvements in task performance.

Given that many concepts in motor learning have unclear or ambiguous definitions, we will define several concepts based on how they are used in this study. “Motor tasks” refer to all tasks that require skilled limb movements to achieve their task goal. Accordingly, most activities of daily living (e.g., cooking, walking, driving) are considered motor tasks even if they engage perceptual, cognitive and motor functions. “Neural processes” refer to brain networks that manipulate perceptual, cognitive and motor information to perform motor tasks. “Motor learning” refers to acquisition and retention of practice-related improvements in task performance, where “task performance” refers to outcomes that are specific to achieving task goals and “improvements” necessitate increased achievement of task goals. We assume that motor learning results from neural adaptations that produce refinements of behavioral features of motor tasks (e.g., skilled limb movements, visual search, eye-hand coordination, visuomotor decisions), where “refinements” are practice-related changes that do not occur in a particular direction.

Traditional studies of motor learning have examined how skilled limb movements are refined during practice of motor tasks [1–3]. Studies of movement dynamics have found that muscle activations, joint torques and endpoint forces exhibit trial-by-trial refinements of coordination and efficiency [4–6]. Similarly, studies of movement kinematics have observed trial-by-trial refinements of speed, accuracy, smoothness and variability of skilled limb movements [7–9], and these refinements exhibit good day-by-day retention [10–13]. However, these studies were not designed to investigate if refinements of other behavioral features, such as visual search, eye-hand coordination and visuomotor decisions, contribute to motor learning.

Research on eye movements indicates that refinements of visual search may contribute to motor learning [14, 15]. Observational studies have found that experts at different visuomotor skills have better control of eye movements than novices [16–20]. Experimental studies have also demonstrated that interventions designed to improve control of eye movements and attention lead to improvements in visuomotor performance [21–25]. While none of these studies examined trial-by-trial or week-by-week refinements of eye movements, there is ample evidence that visual search is refined during practice of perceptual tasks [26–30]. However, these studies did not examine any relationships between refinements of visual search and improvements in task performance, nor did they investigate refinements of other behavioral features. Thus, we do not know if refinements of visual search independently contribute to motor learning.

Studies of spatiotemporal coupling between eye and hand movements have provided evidence that refinements of eye-hand coordination may contribute to motor learning. Patterns of eye-hand coordination vary with task demands [31, 32] and are refined during motor learning in a task-dependent manner [33–36]. However, it remains unclear if refinements of eye-hand coordination independently contribute to improvements in task performance, or if they result from refinements of skilled limb movements and visual search but do not actually contribute to motor learning.

It is widely accepted that sensory processes contribute to planning and execution of skilled limb movements [37]. In addition, information from sensory feedback provides reinforcement that is known to play an important role in motor learning [2]. Recent studies have also found that motor learning can induce changes in visual processing that are associated with refinements of skilled limb movement [38, 39]. This suggests that adaptations of visual and visuomotor processing contribute to motor learning. However, these studies were not designed to investigate the extent to which refinements of other behavioral features, such as visual search, eye-hand coordination and visuomotor decisions, may independently contribute to motor learning.

Despite evidence that refinements of multiple features might underlie motor learning, we do not know the extent to which they independently contribute to motor learning. Traditional experiments cannot easily address this problem because they are designed to isolate individual processes. In contrast, ethological approaches that study real-time, natural behavior can overcome this limitation by leveraging individual patterns of variability exhibited by several behavioral features [40]. However, this approach requires carefully controlling for any covariation between different features. For example, two or more processes may be associated with motor learning, but their individual patterns of variability might exhibit substantial covariance. This shared variance can cause regression analyses to produce incorrect estimates of the contributions made by each process. Accurate estimates of the individual contributions can only be obtained from the independent variance that remains after removing all shared variance.

The objective of the current study was to investigate the extent to which multiple neural processes might independently contribute to motor learning. Healthy young adults used an upper-limb robot with eye tracking to complete six weeks of practice of a novel, visuomotor task designed to mimic the richness of real-world visuomotor tasks. Motor learning was inferred from trial-by-trial acquisition and week-by-week retention of improvements on measures of task performance. Adaptations of multiple neural processes were inferred from trial-by-trial acquisition and week-by-week retention of refinements on measures of skilled limb movement, visual search, eye-hand coordination and visuomotor decisions. Our first hypothesis was that practicing our novel, visuomotor task would elicit trial-by-trial acquisition and week-by-week retention of improvements in task performance that are mirrored by concurrent refinements of skilled limb movements, visual search, eye-hand coordination and visuomotor decisions. Our second hypothesis was that refinements related to multiple neural processes would be independently predictive of improvements in task performance.

## Methods

### Participants

We recruited healthy, young adults (18–35 years old) from the University of South Carolina and surrounding areas. Participants were excluded if they reported any history of a central or peripheral neurological disorder or an ongoing musculoskeletal issue affecting either arm or hand. The study protocol was approved by the University of South Carolina’s Institutional Review Board and all participants provided informed consent to participate.

### Apparatus

**Fig. 1:**
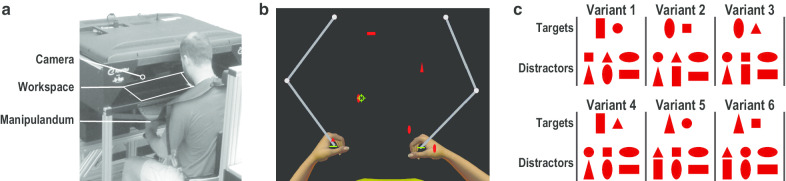
Apparatus and task. **a** Bilateral, upper-limb robot (manipulandum), monocular eye-tracker (camera) and augmented-reality environment (workspace) used for data collection. **b** Overhead view of the Object Hit and Avoid (OHA) task, showing the arms and hands, robotic manipulanda, two green paddles and six red objects (geometric shapes). Participants used the two paddles to hit away 200 target objects and avoid hitting 100 distractor objects that moved toward them from the back of the workspace. The augmented-reality environment presented the paddles and objects in the same horizontal plane as the robotic workspace. Participants were unable to see their arms and hands or the robotic manipulanda. **c** The six OHA variants comprised of six combinations of target objects (one small, one large) and distractor objects (2 small, 4 large)

Data were collected with a bilateral, upper-limb robot (KINARM EndPoint Lab, KINARM, Kingston, Canada) and monocular eye-tracker (EyeLink 1000, SR Research Ltd., Ottawa, Canada) that were integrated with an augmented-reality workspace (Fig. 1a) [41]. Participants sat in a custom chair that used floor-mounted tracks and hydraulics to align them with a forehead rest, which stabilized the head for eye tracking. Participants grasped two near-frictionless manipulanda, which allowed them to make two-dimensional hand movements within an 80 cm wide by 80 cm deep workspace. An opaque shield and fabric cover prevented direct vision of the hands and arms. Hand and gaze position in the robotic workspace were respectively sampled at 1000 and 500 Hz, recorded at 200 Hz, and filtered offline using a low-pass filter with a 20 Hz cutoff.

The augmented-reality environment was created in the same horizontal plane as the robotic workspace by using an inverted-monitor to project visual stimuli at 60 Hz through a semi-transparent mirror. Cartesian gaze position in the horizontal plane was estimated using proprietary calibration algorithms (Kinarm, Kingston, Canada) that provided accurate eye tracking within a workspace of approximately 50 cm wide by 50 cm deep. All visual stimuli were presented within this portion of the robotic workspace. A nonlinear mapping corresponded to a visual area approximately 55° wide by 40° deep in which stimuli located closer to participants comprised larger visual angles.

### Task

Participants practiced six trials of a continuous, visuomotor task, Object Hit and Avoid (OHA) [42], once a week for six consecutive weeks. Each participant was scheduled at a consistent time of day on the same weekday to avoid potential confounds caused by circadian rhythms and to assure a consistent retention interval between sessions. Illumination of the room was maintained at a constant level for the duration of the study. 

In each trial of the OHA task, 300 red objects comprised of eight geometric shapes (e.g., square, circle, triangle, etc.) moved from the back of the workspace towards the participants along ten parallel paths (5 cm center-to-center spacing) (Fig. 1b). Two shapes were predefined as “Targets” and six shapes were predefined as “Distractors”. Each parallel path contained 20 Targets (n = 200) and 10 Distractors (n = 100) that were released in random order. The average number of objects that were simultaneously present in the workspace and the average speed that objects moved progressively increased over time. As a result, task difficulty increased within each trial, whereas the overall difficulty of each trial was consistent. Each trial ended after all 300 objects had passed through the workspace (~ 2 min).

Participants received standardized instructions to use two green paddles (2.5 cm wide) located on top of each hand to hit away as many Targets and to avoid hitting as many Distractors as possible. When participants made paddle contact with Targets, the robot applied a small perturbation (10 Newtons for 50 ms) to the participant’s hand and Targets rebounded from the paddle with the same direction and speed as the paddle movement. When participants made paddle contact with Distractors, no perturbation was applied to the participant’s hand and Distractors passed unaltered through the paddle. Paddle size, object size and the spacing between adjacent paths prevented participants from simultaneously hitting two objects with the same hand.

We employed six distinct variants of targets and distractors to prevent overlearning of a specific variant from causing plateaus in task performance (Fig. 1c). Each variant was pseudo-randomized and counter-balanced between participants each week and was never practiced by a participant in more than one week. Specifically, each of the six variants was assigned to three different participants each week, and each participant performed six trials of a different variant each week. Before starting each trial, the two target shapes were presented in the middle of workspace until participants confirmed that they had memorized the shapes and were ready to begin. After each trial, participants were offered a rest period until they were ready to start the next trial.

### Gaze classification

Gaze data were processed and classified using the procedures of a validated methodology for processing gaze data our group previously published [41]. In brief, the methodology involves preprocessing gaze data to remove blink artifacts, one sample spikes caused by incorrect corneal detection, and outliers that occurred when gaze moved outside the eye-tracking workspace. We subsequently use a novel geometric method to transform gaze position data into rotational kinematics of the eye. Finally, we use adaptive thresholding methods to classify eye movements into saccades (rapid eye movements between targets) and smooth pursuits (eye movements that followed moving targets with foveal vision). Our previous manuscript demonstrated that our methodology for gaze processing and classification correctly classifies approximately 90% of saccades and smooth pursuits and misclassifies approximately 5% of saccades and smooth pursuits when compared with manual classification (gold standard) [41].

### Measures

We used hand and gaze data to compute measures of Task Performance, Skilled Limb Movement, Visual Search, Eye-Hand Coordination and Visuomotor Decisions for each OHA trial.

*Task performance *We computed two measures of task performance (Eqs. 1 and 2). Targets Hit (%) quantified goal achievement resulting from successful execution of hand movements to hit targets (motor execution). It was calculated as the percent of all 200 targets that participants “hit”, where a target was counted as “hit” if either paddle made contact with the target, causing it to move toward the back of the workspace. Only one “hit” was counted if a target was hit more than once. Distractors Avoided (%) quantified goal achievement resulting from successful inhibition of hand movements to avoid distractors. It was calculated as the percent of all 100 Distractors that were “not hit”, where a distracter was counted as “not hit” if neither paddle made contact with the distractor or if a paddle made contact but caused the distractor to move toward the front of the workspace.

1 $$Targets Hit=\frac{{N}_{Targets Hit}}{200 Targets} * 100\mathrm{\%}$$

2 $$Distractors Avoided=\frac{{N}_{Distractors Not Hit}}{100 Distractors} * 100\mathrm{\%}$$

*Skilled limb movement *We computed five measures of skilled limb movement (Eqs. 3–7). Mean Hand-Speed (cm/s) quantified the overall execution speed of all hand movements by computing the average speed of right- and left-hand movements. Mean Hand-Area (cm^2^) quantified the overall spatial distribution of all hand movements by calculating the average area covered by right- and left-hand movements, where each area was obtained by computing the convex hull of left- and right-hand movements. Target Contact Speed (cm/s) quantified the execution speed of skilled hand movements by computing the average speed of hand movements at the onset of paddle-contact with each target that was successfully hit. Hand-Speed Bias quantified bimanual coordination by computing inter-limb differences in movement speed. It was calculated as the normalized difference between the average speed of right- and left-hand movements. Hand-Area Bias quantified bimanual coordination by computing inter-limb differences in the spatial distributions of hand movements. It was calculated as the normalized difference between the area covered by movements of the right and left hands. Values of hand-speed bias or hand-area bias near zero indicate equal use of both hands and increasingly higher values indicate greater use of one hand than the other. We were unable to quantify many traditional measures of skilled limb movement, such as time to peak velocity, peak acceleration or smoothness, because we could not identify a distinct start or end point of most limb movements due to the continuous nature of our task.

3 $$Mean Hand-Speed=\frac{\stackrel{-}{{Hand-Speed}_{Right}} + \stackrel{-}{{Hand-Speed}_{Left}}}{2 Hands}$$

4 $$Mean Hand-Area=\frac{{Hand-Area}_{Right} + {Hand-Area}_{Left}}{2 Hands}$$

5 $$Hand-Speed Bias=\left|\frac{\stackrel{-}{{Hand-Speed}_{Right}} - \stackrel{-}{{Hand-Speed}_{Left}}}{\stackrel{-}{{Hand-Speed}_{Right}} + \stackrel{-}{{Hand-Speed}_{Left}}}\right|$$

6 $$Hand-Area Bias=\left|\frac{{Hand-Area}_{Right} - {Hand-Area}_{Left}}{{Hand-Area}_{Right} + {Hand-Area}_{Left}}\right|$$

7 $$Target Contact Speed=\frac{{\sum }_{1}^{N}{Hand-Speed}_{Target Contact}}{{N}_{Targets Hit}}$$

*Visual search *We computed three measures of visual search (Eqs. 8–10). Objects Foveated (%) quantified the overall efficiency of visual search by calculating the percent of all 300 objects that participants “foveated” with pursuit eye movements, where an object was counted as “foveated” if the object was followed with foveal vision for at least 40 ms [41]. If an object was foveated more than once, it was only counted once. Spatial Foveation Bias quantified spatial biases in the distribution of visual search by computing the normalized difference between the number of objects foveated on the right and left sides of the workspace. Extrafoveal Hits (%) quantified covert use of parafoveal and peripheral vision for visual search by calculating the percent of targets that were hit but were not previously foveated. We were unable to compute other measures of visual search because a large number of catch-up saccades during pursuit prevented accurate calculation of other valid measures.

8 $$Objects Foveated=\frac{{N}_{Objects Fov}}{300 Objects} * 100\mathrm{\%}$$

9 $$Spatial Foveation Bias=\left|\frac{{N}_{Objects Fov on Right} - {N}_{Objects Fov on Left}}{{N}_{Objects Fov on Right} + {N}_{Objects Fov on Left}}\right|$$

10 $$Extrafoveal Hits=\frac{{N}_{Targets Hit \cap Not Foveated}}{{N}_{Targets Not Foveated}}*100\mathrm{\%}$$

*Eye–hand coordination *We computed two measures of eye-hand coordination (Eqs. 11–12). Gaze-Hand Distance (cm) quantified spatial coupling between the eyes and hands by calculating the distance between gaze and hand position at the onset of paddle-contact with each target [33]. Gaze-Hand Latency (ms) quantified temporal coupling between eyes and hands by calculating the interval between the initial time of each target hit and final time that gaze foveated the target [33–36, 43]. If a target was hit more than once, only the first hit was included in these calculations. If a target was not foveated or was hit before it was foveated, it was excluded from these calculations.

11 $$Gaze-Hand Distance=\frac{{\sum }_{1}^{N}\sqrt{({X}_{Gaze} - {X}_{Target}{)}^{2} + ({Y}_{Gaze} - {Y}_{Target}{)}^{2}}}{{N}_{Targets Hit}}$$

12 $$Gaze-Hand Latency=\frac{{\sum }_{1}^{N}({Time}_{Initial Contact } - {Time}_{Final Fov})}{{N}_{Targets Hit}}$$

*Visuomotor decisions *We computed three measures of visuomotor decisions (Eqs. 13–15). Target Foveation Time (ms) quantified the amount of time used for making decisions to hit targets and was calculated as the average duration that participants foveated targets. Distractor Foveation Time (ms) quantified the amount of time used for making decisions to avoid distractors and was calculated as the average duration that participants foveated distractors. If a target or distractor was foveated more than one time, we included the total time of all foveations. Both measures quantified the average time used to recognize and classify shapes as a target or distractor. However, Target Foveation Time included the average time used to initiate hand movements, whereas Distractor Foveation Time included the average time used to inhibit hand movements. Foveation Time Difference (ms) quantified differences between the amount of time used for making decisions to hit targets and avoid distractors and was calculated as the difference between target and distractor foveation times.

13 $$Target Foveation Time=\frac{{\sum }_{1}^{N}Target Fov Time}{{N}_{Targets Foveated}}$$

14 $$Distractor Foveation Time=\frac{{\sum }_{1}^{N}Distractor Fov Time }{{N}_{Distractors Foveated}}$$

15 $$Fov Time Diff=Target Foveation Time-Distractor Foveation Time$$

### Analysis

All analyses were performed using Matlab 2017b (Mathworks Inc., Natick, MA).

#### *Validationofmeasures*

Since most of our measures were novel, we first examined each measure for uniqueness of information and for the presence of outliers. We confirmed that each measure quantified unique information by examining the covariance between each pair of measures. If we found a moderate Pearson correlation coefficient between any pair of measures (|*r*|≥ 0.707, *r*^2^ ≥ 0.5), we excluded the measure with the highest coefficient of variance from further analyses [44]. We subsequently performed a visual inspection of our data, which revealed the presence of a small number of outliers in several measures. For all subsequent analyses, we minimized the potential influence of outliers by performing robust regression with a Welsch weighting function [45]. Finally, we standardized each measure to obtain a mean of zero and standard deviation of one, which allowed us to compare measures with different units.

#### *Practice-relatedrefinements*

**Fig. 2:**
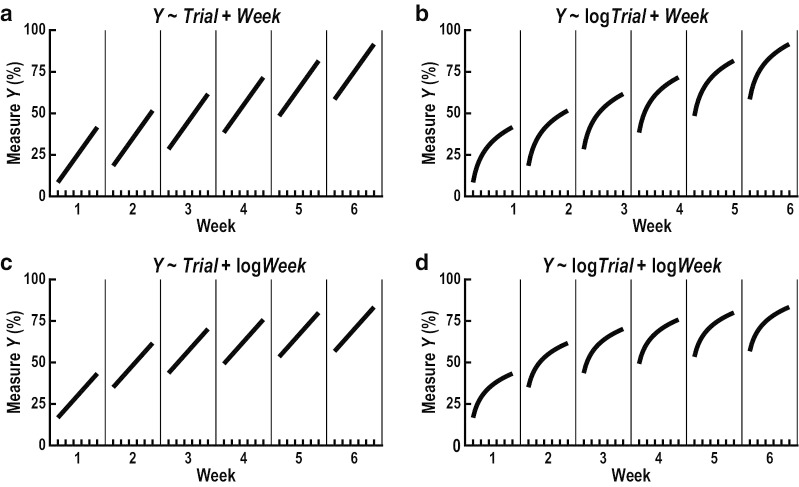
Theoretical models used to quantify trial-by-trial acquisition and week-by-week retention of refinements. **a** Linear trial-by-trial and linear week-by-week refinements. **b** Logarithmic trial-by-trial and linear week-by-week refinements. **c** Linear trial-by-trial and logarithmic week-by-week refinements. **d** Logarithmic trial-by-trial and logarithmic week-by-week refinements

Our first hypothesis was that practice would induce trial-by-trial and week-by-week refinements of skilled limb movement, visual search, eye-hand coordination and visuomotor decisions that mirror improvements in task performance. We tested this hypothesis by using robust regression to compare eight different linear mixed-effects models that quantified trial-by-trial acquisition and week-by-week retention of refinements (Eqs. 16–23). The first four models implemented different combinations of linear and logarithmic growth rates (linear–linear, linear–logarithmic, logarithmic–linear, logarithmic–logarithmic) to quantify trial-by-trial acquisition and week-by-week retention of refinements (Fig. 2). The other four models added an interaction term that quantified trial-by-trial changes across weeks.

16 $${Y}_{ijk}={b}_{i}+{\beta }_{1}{Trial}_{j}+{\beta }_{2}{Week}_{k}+{\epsilon }_{ijk}$$

17 $${Y}_{ijk}={b}_{i}+{\beta }_{1}\mathrm{log}{Trial}_{j}+{\beta }_{2}{Week}_{k}+{\epsilon }_{ijk}$$

18 $${Y}_{ijk}={b}_{i}+{\beta }_{1}{Trial}_{j}+{\beta }_{2}\mathrm{log}{Week}_{k}+{\epsilon }_{ijk}$$

19 $${Y}_{ijk}={b}_{i}+{\beta }_{1}\mathrm{log}{Trial}_{j}+{\beta }_{2}\mathrm{log}{Week}_{k}+{\epsilon }_{ijk}$$

20 $${Y}_{ijk}={b}_{i}+{\beta }_{1}{Trial}_{j}+{\beta }_{2}{Week}_{k}+{\beta }_{3}({Trial}_{j}*{Week}_{k})+{\epsilon }_{ijk}$$

21 $${Y}_{ijk}={b}_{i}+{\beta }_{1}\mathrm{log}{Trial}_{j}+{\beta }_{2}{Week}_{k}+{\beta }_{3}(\mathrm{log}{Trial}_{j}*{Week}_{k})+{\epsilon }_{ijk}$$

22 $${Y}_{ijk}={b}_{i}+{\beta }_{1}{Trial}_{j}+{\beta }_{2}\mathrm{log}{Week}_{k}+{\beta }_{3}\left({Trial}_{j}*\mathrm{log}{Week}_{k}\right)+{\epsilon }_{ijk}$$

23 $${Y}_{ijk}={b}_{i}+{\beta }_{1}\mathrm{log}{Trial}_{j}+{\beta }_{2}\mathrm{log}{Week}_{k}+{\beta }_{3}(\mathrm{log}{Trial}_{j}*\mathrm{log}{Week}_{k})+{\epsilon }_{ijk}$$

In Eqs. 16–23, ${Y}_{ijk}$ represents each measure obtained from participant $i$, in trial $j$ of week $k$, ${b}_{i}$ is a random intercept for each participant, ${\beta }_{1}$ describes trial-by-trial acquisition of refinements, ${\beta }_{2}$ describes week-by-week retention of refinements, and ${\epsilon }_{ijk}$ is the error term. In Eqs. 20–23, ${\beta }_{3}$ is an interaction term that describes changes in trial-by-trial refinements across weeks. The model with the lowest Bayesian Information Criterion (BIC) was used to examine trial-by-trial acquisition and week-by-week retention of refinements. After finding the best-fit model for each measure, we verified that additional transformations were not required by visually inspecting the fit between the predicted and actual outcomes and by testing the residuals for normality with Kolmogorov-Smirnoff tests. Measures with at least a small effect size (${f}^{2}$≥0.02; [46] for trial-by-trial acquisition (${\beta }_{1}$) or week-by-week retention (${\beta }_{2}$) of refinements were subsequently included as “predictor measures” in the following analyses of our second hypothesis.

#### *Predictionofmotorlearning*

Our second hypothesis was that refinements related to multiple neural processes would be independently predictive of improvements in task performance. We tested this hypothesis by using multiple regression to quantify the extent to which refinements of predictor measures were independently predictive of improvements on our two measures of task performance (outcome measures). Before performing these multiple regression analyses, we first reduced the number of predictor measures included in each model by using bivariate regression to confirm that each predictor measure that was individually related to improvements on our two measures of task performance (i.e., at least a small effect size, ${f}^{2}$≥0.02). We then examined each predictor measure for multicollinearity by computing the Tolerance of each measure, which is the proportion of variance not explained by linear combinations of all other predictors (i.e., 1–${R}^{2}$) [47]. We subsequently performed multiple regression using linear mixed-effects models that only included the predictor measure identified in the previous step (Eq. 24).

24 $${Y}_{ijk}={b}_{i}+{\beta }_{1}{X}_{1(ijk)}+{\beta }_{2}{X}_{2(ijk)}+\dots +{\beta }_{N}{X}_{n(ijk)}+{\epsilon }_{ijk}$$

In Eq. 24, ${Y}_{ijk}$ represents task performance of participant $i$ in trial $j$ of week $k$, ${b}_{i}$ are random intercepts for each participant, coefficients ${\beta }_{1}$–${\beta }_{N}$ are estimated relationships between each predictor measure (${X}_{1}$–${X}_{n}$) and the respective measure of task performance, and ${\epsilon }_{ijk}$ is the error term.

**Fig. 3:**
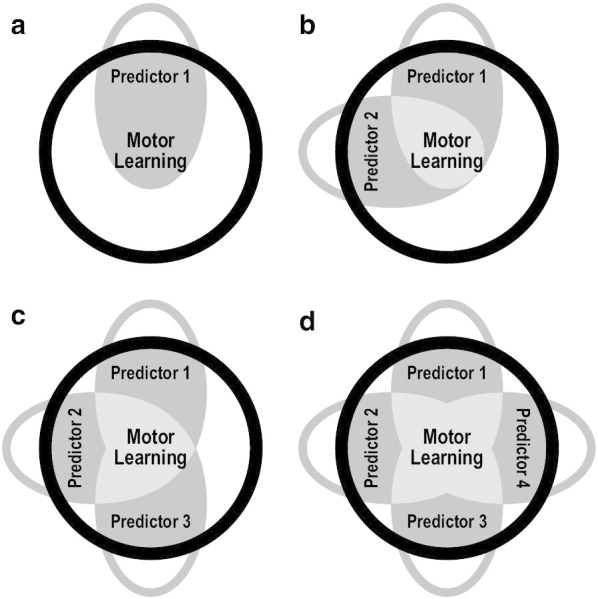
Conceptual illustrations of regression analyses used to examine motor learning. **a** Diagram showing how bivariate regression quantifies relationships between an individual predictor and motor learning without removing the variance shared with other potential predictors. **b**–**d.** Diagrams showing how multiple regression quantifies relationships between two (**b**), three (**c**) or four (**d**) predictors and motor learning. Regression coefficients estimate relationships from the independent and shared variance of each predictor, whereas semipartials estimate relationships from only the independent variance of each predictor. Light grey areas show portions of motor learning that cannot be attributed to a single predictor due to shared variance with other predictors. Dark grey areas show portions of motor learning that can be attributed to a single predictor after removing its shared variance

We finally identified each predictor measure that was independently predictive of improvements on our two measures of task performance. Importantly, the values of coefficients ${\beta }_{1}$–${\beta }_{N}$ in Eq. 24 are influenced by variance that is independent of all other predictors and variance that is shared with other predictors. Figure 3 illustrates conceptual representations of independent and shared variance for four theoretical regression models that include one, two, three, or four predictors of motor learning. If only one predictor is examined (a), it might be assumed that all variance related to motor learning (dark grey area) is independently predictive of motor learning. However, if multiple predictors are examined (b–d), part of each predictor’s variance related to motor learning would be independent of all other predictors (dark grey area) and part would be shared with other predictors (light grey area). The relationships between each predictor’s independent variance and motor learning are described by semipartial coefficients of determination (${sr}^{2}$). For the purpose of our second hypothesis, we calculated semipartial coefficients of determination (${sr}^{2}$), semipartial effect sizes (${sf}^{2}$), and semipartial *p*-values ($sp$) to examine the relationships between the independent variance of each predictor measure and improvements on our two measures of task performance. We considered measures with at least a small semipartial effect size (${sf}^{2}$≥0.02) as meaningful predictors of motor learning, though we recognize that this could underestimate the amount of motor learning that should be attributed to each predictor.

For the purpose of rigor and reproducibility, we validated our multiple regression results by performing forward and backward stepwise regression with the same set of predictor measures used in our multiple regression analyses. We used the BIC to determine which predictor to add or remove at each step. This resulted in a final model with a minimum BIC.

## Results

### Participants

We enrolled 18 healthy, young adults (8 male, 10 female; 24.2 ± 3.7 years old; 17 R-handed, 1 L-handed) in the study. One participant was unable to complete the sixth week of the study. We included the participant’s data without replacement of the sixth week.

### Exemplar OHA performance

**Fig. 4:**
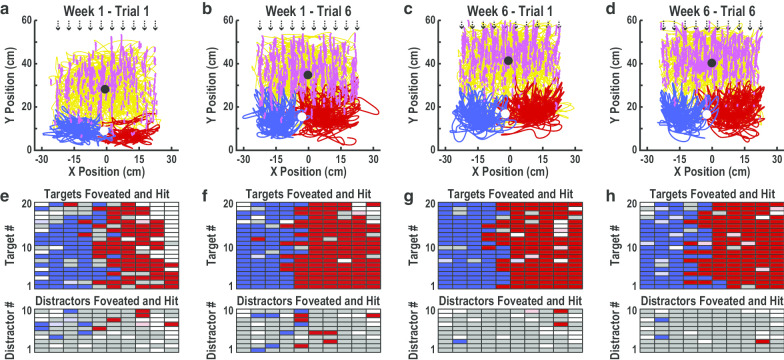
Eye and hand movements and target/distractor foveations and hits by an exemplar participant during two trials on Week 1 and two trials on Week 6. **a**–**d.** *X* position (width) versus *Y* position (depth) of eye and hand movements on Week_1_·Trial_1_ (**a**), Week_1_·Trial_6_ (**b**), Week_6_·Trial_1_ (**c**), and Week_6_·Trial_6_ (**d**). Colored lines illustrate pursuit eye movements (pink), saccadic eye movements (gold), left-hand movements (blue) and right-hand movements (red). Dashed arrows indicate the ten parallel paths that objects moved along. Black and white circles show the Center-of-Mass of gaze and hand movements, respectively. **e**–**h** Task performance on Week_1_·Trial_1_ (**e**), Week_1_·Trial_6_ (**f**), Week_6_·Trial_1_ (**g**) and Week_6_·Trial_6_ (**h**). The upper grids (20 × 10) represent each target and the lower grids (10 × 10) represent each distractor that was foveated and hit with the left hand (dark blue), foveated and hit with the right hand (dark red), foveated but not hit (grey), not foveated but hit with the left hand (light blue), not foveated but hit with the right hand (light red), or neither foveated nor hit (white)

Figure 4 illustrates pursuit and saccadic eye movements (pink and gold lines) and left- and right-hand movements (blue and red lines) made by an exemplar participant at four time points, Week_1_·Trial_1_ (a), Week_1_·Trial_6_ (b), Week_6_·Trial_1_ (c), and Week_6_·Trial_6_ (d). At each time point, the participant’s eye movements covered an area of approximately 50 cm wide (*X*) by 40 cm deep (*Y*). The center-of-mass was consistently located near the midline but shifted distally from around 30 cm on Week_1_·Trial_1_ (a), to 35 cm on Week_1_·Trial_6_ (b), and 40 cm on Week_6_·Trial_1_ and Week_6_·Trial_6_ (c, d). Combined movements of both hands covered an area that was around 50 cm wide and consistently centered near the midline. However, the range of hand movements in depth increased from around 15 cm on Week_1_·Trial_1_ (a) to 20 cm on the other three trials (b–d). The center-of-mass also shifted distally from under 10 cm on Week_1_·Trial_1_ (a) to over 15 cm on the other three trials (b–d). Left- and right-hand movements covered similar areas and were largely constrained to their respective sides.

Figure 4 also displays grids of rectangles that represent each Target (upper grids: 20 × 10) and Distractor (lower grids: 10 × 10) that was foveated and hit (left hand: dark blue, right hand: dark red), foveated but not hit (grey), not foveated but hit (left hand: light blue, right hand: light red), or neither foveated nor hit (white). The participant failed to foveate several targets and distractors on Week_1_·Trial_1_ (e) but foveated the majority of targets and distractors on the other three trials (f–h). Similarly, the participant failed hit a number of targets on Week_1_·Trial_1_ (e) but hit the majority of targets on the other three trials (f–h). In contrast, the participant hit several distractors in the first week (e, f) but very few in the last week (f–h). At all four time points, the participant hit more targets with the right-hand, including several targets on the left side of the workspace.

### Validation of measures

Targets Hit and Distractors Avoided exhibited a low correlation ($r$= 0.03), indicating that they quantified unique aspects of task performance. Both measures were included in our subsequent analyses. We also examined each pair of predictor measures for high correlations ($|r|$≥ 0.707) indicative of redundant information (Table 1). Two pairs exhibited high correlations, Mean Hand-Speed and Target Contact Speed ($r$= 0.89) and Gaze-Hand Distance and Gaze-Hand Latency ($r$= 0.89). Target Contact Speed and Gaze-Hand Latency were excluded from all remaining analyses because they had the highest coefficients of variance in each pair [44].

**Table 1:** Coefficients of variance and correlations between measures

Measure	Coefficient of variance	Correlation matrix												
		MHS	MHA	TCS	HSB	HAB	OF	SFB	EH	GHD	GHL	TFT	DFT	FTD
Mean Hand-Speed (MHS)	0.30	1												
Mean Hand-Area (MHA)	0.29	− 0.58	1											
Target Contact Speed (TCS)	**0.44**	− **0.89**	− 0.35	1										
Hand-Speed Bias (HSB)	0.59	− 0.11	− 0.06	− 0.08	1									
Hand-Area Bias (HAB)	0.68	− 0.02	− 0.07	− 0.05	− 0.67	1								
Objects Foveated (OF)	0.09	− 0.12	− 0.15	− 0.03	− 0.04	− 0.01	1							
Spatial Foveation Bias (SFB)	0.93	− 0.08	− 0.05	− 0.02	− 0.01	− 0.16	− 0.07	1						
Extrafoveal Hits (EH)	0.58	− 0.09	− 0.07	− 0.06	− 0.05	− 0.08	− 0.13	− 0.21	1					
Gaze-Hand Distance (GHD)	0.28	− 0.06	− 0.32	− 0.12	− 0.05	− 0.09	− 0.13	− 0.09	− 0.19	1				
Gaze-Hand Latency (GHL)	**0.47**	− 0.26	− 0.39	− 0.06	− 0.11	− 0.08	− 0.03	− 0.05	− 0.20	− **0.89**	1			
Target Foveation Time (TFT)	0.12	− 0.32	− 0.27	− 0.22	− 0.12	− 0.11	− 0.31	− 0.19	− 0.33	− 0.14	− 0.09	1		
Distractor Foveation Time (DFT)	0.16	− 0.16	− 0.16	− 0.01	− 0.08	− 0.08	− 0.20	− 0.14	− 0.22	− 0.11	− 0.12	− 0.66	1	
Foveation Time Difference (FTD)	0.36	− 0.18	− 0.11	− 0.24	− 0.04	− 0.03	− 0.12	− 0.05	− 0.12	− 0.30	− 0.26	− 0.36	− 0.46	1

### Confirmation of motor learning

**Fig. 5:**
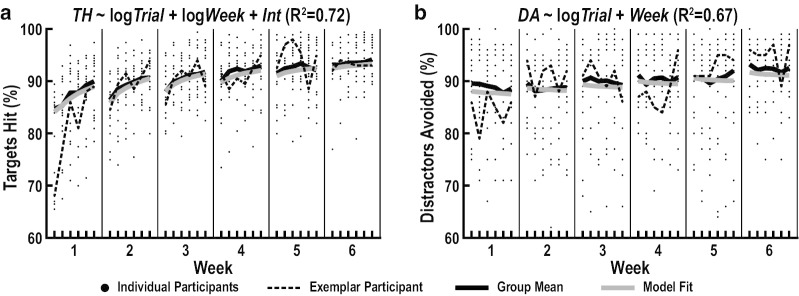
Improvements on measures of task performance. Trial-by-trial acquisition and week-by-week retention of improvements on Targets Hit (**a**) and Distractors Avoided (**b**). Each panel shows raw data values of all individual participants (small black dots), raw data values of the exemplar participant in Fig. 4 (thin dashed lines), group means of the reweighted participant data (thick black lines), and model predictions (thick grey lines). Reweighted participant data were obtained by applying weights from the robust regression to the raw data values of all participants. The model with the best overall fit (lowest Bayesian Information Criterion) is displayed at the top of each panel

Before testing our two hypotheses, we first confirmed that our participants demonstrated trial-by-trial acquisition and week-by-week retention of improvements in task performance (Table 2). Targets Hit exhibited moderate trial-by-trial increases (${\beta }_{1}$=0.26, ${f}^{2}$=0.23, $p$<10^–6^), large week-by-week increases (${\beta }_{2}$=0.49, ${f}^{2}$=0.82, $p$<10^–6^), and small trial-by-trial decreases across weeks (${\beta }_{3}$=-0.16, ${f}^{2}$=0.09, $p$<10^–6^) (Fig. 5a). We also observed small, week-by-week increases on Distractors Avoided (${\beta }_{2}$=0.20, ${f}^{2}$=0.11, $p$<10^–6^) (Fig. 5b). These finding show that practice-related improvements in motor execution (Targets Hit) and motor inhibition (Distractors Avoided) contributed to improvements in task performance.

**Table 2:** Practice-related improvements of outcome and predictor measures

Category	Measure	Trial × Trial						Week × Week						Interaction				
		Fit	${\beta }_{1}$	SE	${r}^{2}$	${f}^{2}$	$p$	Fit	${\beta }_{2}$	SE	${r}^{2}$	${f}^{2}$	$p$	${\beta }_{3}$	SE	${r}^{2}$	${f}^{2}$	$p$
Task	TH	log	0.26	0.02	0.07	**0.23**	< 10^–6^	log	0.49	0.02	0.24	**0.82**	< 10^–6^	− 0.16	0.02	0.03	0.09	< 10^–6^
	DA	log	− 0.13	0.02	0.00	0.00	0.17	ln	0.20	0.02	0.04	**0.11**	< 10^–6^					
Skilled Limb Movement	MHS	log	0.09	0.02	0.01	**0.03**	< 10^–4^	ln	0.21	0.02	0.04	**0.14**	< 10^–6^					
	MHA	log	0.07	0.03	0.00	0.01	0.01	ln	0.07	0.03	0.00	0.01	0.009	− 0.08	0.03	0.01	0.01	0.004
	HSB	ln	0.07	0.03	0.01	0.01	0.01	ln	0.14	0.03	0.02	**0.03**	< 10^–5^	0.08	0.03	0.01	0.01	0.005
	HAB	ln	0.14	0.03	0.02	**0.03**	< 10^–4^	ln	0.04	0.03	0.00	0.00	0.19					
Visual Search	OF	log	0.12	0.02	0.01	**0.04**	< 10^–6^	log	0.32	0.02	0.10	**0.29**	< 10^–6^	− 0.12	0.02	0.01	0.04	< 10^–6^
	EH	log	0.22	0.03	0.05	**0.12**	< 10^–6^	ln	0.38	0.03	0.15	**0.36**	< 10^–6^					
	SFB	log	0.04	0.03	0.00	0.00	0.22	ln	0.10	0.03	0.01	0.01	0.001					
Eye-Hand	GHD	log	− 0.01	0.02	0.00	0.00	0.65	log	0.33	0.02	0.11	**0.69**	< 10^–6^	− 0.07	0.02	0.01	0.03	< 10^–5^
Visuomotor Decisions	TFT	log	− 0.06	0.02	0.00	0.01	0.01	log	− 0.27	0.02	0.08	**0.20**	< 10^–6^	0.11	0.02	0.01	0.03	< 10^–5^
	DFT	ln	0.02	0.02	0.00	0.00	0.33	ln	0.11	0.02	0.01	**0.04**	< 10^–6^	0.08	0.02	0.01	0.02	0.001
	FTD	log	− 0.10	0.03	0.01	0.01	0.002	log	− 0.41	0.03	0.16	**0.25**	< 10^–6^					

Bold indicates measures that exhibited meaningful trial-by-trial or week-by-week changes (*f*^2^ ≥ 0.02).

*SE* standard error, *TH* Targets Hit, *DA* Distractors Avoided, *MHS* Mean Hand-Speed, *MHA* Mean Hand-Area, *HSB* Hand-Speed Bias, *HAB* Hand-Area Bias, *OF* Objects Foveated, *EH* Extrafoveal Hits, *SFB* Spatial Foveation Bias, *GHD* Gaze-Hand Distance, *TFT* Target Foveation Time, *DFT* Distractor Foveation Time, *FTD* Foveation Time Difference

### Practice-related refinements

**Fig. 6:**
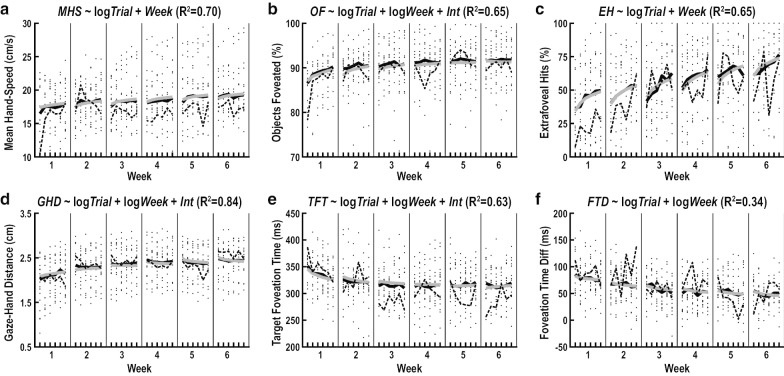
Refinements on measures of behavioral features. Trial-by-trial acquisition and week-by-week retention of refinements on Mean Hand-Speed (**a**), Objects Foveated (**b**), Extrafoveal Hits (**c**), Gaze-Hand Distance (**d**), Target Foveation Time (**e**) and Foveation Time Difference (**f**). Each panel shows raw data values of all individual participants (small black dots), raw data values of the exemplar participant in Fig. 4 (thin dashed lines), group means of the reweighted participant data (thick black lines), and model predictions (thick grey lines). Reweighted participant data were obtained by applying weights from the robust regression to the raw data values of all participants. The model with the best overall fit (lowest Bayesian Information Criterion) is displayed at the top of each panel

We tested our first hypothesis by examining trial-by-trial acquisition and week-by-week retention of refinements on our measures of skilled limb movement, visual search, eye-hand coordination and visuomotor decisions (Table 2). Three measures of skilled limb movement (Mean Hand-Speed, Hand-Speed Bias, and Hand-Area Bias) displayed practice-related refinements. Mean Hand-Speed exhibited small trial-by-trial increases (${\beta }_{1}$=0.09, ${f}^{2}$=0.03, $p$<10^–4^) and small week-by-week increases (${\beta }_{2}$=0.21, ${f}^{2}$=0.14, $p$<10^–6^) (Fig. 6a). Hand-Speed Bias demonstrated small week-by-week increases (${\beta }_{2}$=0.14, ${f}^{2}$=0.03, $p$<10^–5^) and Hand-Area Bias showed small trial-by-trial increases (${\beta }_{1}$=0.14, ${f}^{2}$=0.03, $p$<10^–4^). Two measures of visual search (Objects Foveated and Extrafoveal Hits) exhibited practice-related refinements. Objects Foveated displayed small trial-by-trial increases (${\beta }_{1}$=0.12, ${f}^{2}$=0.04, $p$<10^–6^), moderate week-by-week increases (${\beta }_{2}$=0.32, ${f}^{2}$=0.29, $p$<10^–6^), and small trial-by-trial decreases across weeks (${\beta }_{3}$=− 0.12, ${f}^{2}$=0.04, $p$<10^–6^) (Fig. 6b). Extrafoveal Hits exhibited small trial-by-trial increases (${\beta }_{1}$=0.22, ${f}^{2}$=0.12, $p$<10^–6^) and large week-by-week increases (${\beta }_{2}$=0.38, ${f}^{2}$=0.36, $p$<10^–6^) (Fig. 6c). Our only measure of eye-hand coordination, Gaze-Hand Distance, demonstrated large week-by-week increases (${\beta }_{2}$=0.33, ${f}^{2}$=0.69, $p$<10^–6^) (Fig. 6d). All three measures of visuomotor decisions (Target Foveation Time, Distractor Foveation Time and Foveation Time Difference) displayed practice-related refinements. Target Foveation Time showed moderate week-by-week decreases (${\beta }_{2}$=− 0.27, ${f}^{2}$=0.20, $p$<10^–6^) (Fig. 6e). Distractor Foveation Time displayed small week-by-week increases (${\beta }_{2}$=0.11, ${f}^{2}$=0.04, $p$<10^–6^). Foveation Time Difference exhibited moderate week-by-week decreases (${\beta }_{2}$=− 0.41, ${f}^{2}$=0.25, $p$<10^–6^) (Fig. 6f). One measure of skilled limb movement (Mean Hand-Area) and one measure of visual search (Spatial Foveation Bias) did not exhibit practice-related refinements and were excluded from further analyses.

### Prediction of motor learning

**Fig. 7:**
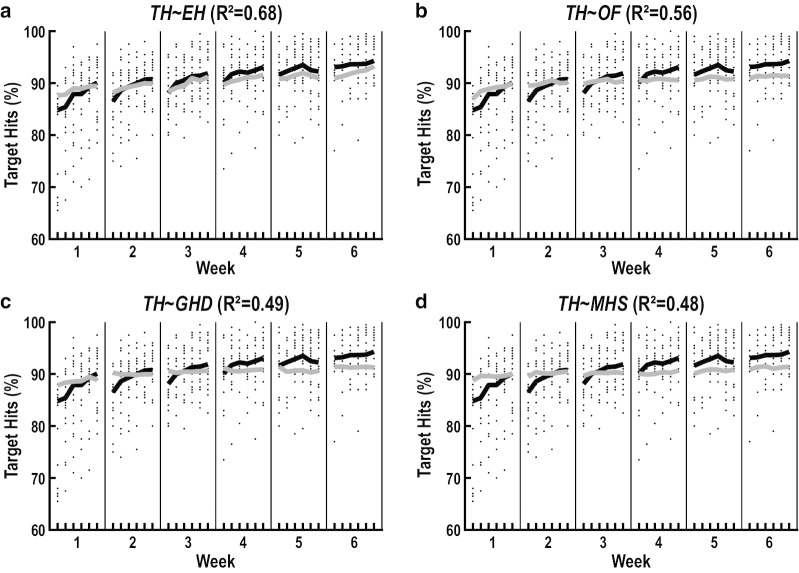
Univariate predictions of task performance. Illustrations show the predicted improvements in Targets Hit obtained from bivariate regression models using Extrafoveal Hits (**a**), Objects Foveated (**b**), Gaze-Hand Distance (**c**) and Mean Hand-Speed (**d**) as predictors. Each panel shows raw data values of all individual participants (small black dots), group means of the reweighted participant data (thick black lines), and model predictions (thick grey lines). Reweighted participant data were obtained by applying weights from the robust regression to the raw data values of all participants

We initially used bivariate regression to identify predictor measures that were individually related to improvements on our two measures of task performance (i.e., at least a small effect size, ${f}^{2}$≥0.02) (Table 3). We identified six predictor measures that were individually related to improvements in Targets Hit. They included Extrafoveal Hits ($\beta$=0.70, ${f}^{2}$=1.52, $p$<10^–6^) (Fig. 7a), Objects Foveated ($\beta$=0.59, ${f}^{2}$=0.80, $p$<10^–6^) (Fig. 7b), Gaze-Hand Distance ($\beta$=0.58, ${f}^{2}$=0.65, $p$<10^–6^) (Fig. 7c), Mean Hand-Speed ($\beta$=0.50, ${f}^{2}$=0.48, $p$<10^–6^) (Fig. 7d), Target Foveation Time ($\beta$=-0.46, ${f}^{2}$=0.41, $p$<10^–6^), and Foveation Time Difference ($\beta$=0.23, ${f}^{2}$=0.09, $p$<10^–6^). We also identified six predictor measures that were individually related to improvements in Distractors Avoided. They included Gaze-Hand Distance ($\beta$=0.29, ${f}^{2}$=0.25, $p$<10^–6^), Target Foveation Time ($\beta$=− 0.11, ${f}^{2}$=0.04, $p$<10^–3^), Hand-Speed Bias ($\beta$=0.11, ${f}^{2}$=0.03, $p$<10^–3^), Extrafoveal Hits ($\beta$=0.11, ${f}^{2}$=0.03, $p$<10^–3^), Foveation Time Difference ($\beta$=− 0.09, ${f}^{2}$=0.02, $p$<10^–3^), and Objects Foveated ($\beta$=0.09, ${f}^{2}$=0.02, $p$<0.01).

**Table 3:** Bivariate regression between predictor and outcome measures

Outcome measures	Predictor measures	$\beta$	SE	${r}^{2}$	${f}^{2}$	$p$	$p$_(residuals)_
Targets Hit	Extrafoveal Hits	− 0.70	0.03	< 0.49	< **1.52**	< 10^–6^	0.07
	Objects Foveated	− 0.59	0.04	< 0.35	< **0.80**	< 10^–6^	0.27
	Gaze-Hand Distance	− 0.58	0.05	< 0.33	< **0.65**	< 10^–6^	0.31
	Mean Hand-Speed	− 0.50	0.05	< 0.25	< **0.48**	< 10^–6^	0.07
	Target Foveation Time	− 0.46	0.04	< 0.21	< **0.41**	< 10^–6^	0.14
	Foveation Time Difference	− 0.23	0.03	< 0.05	< **0.09**	< 10^–6^	0.05
	Hand-Speed Bias	− 0.10	0.04	< 0.01	< 0.02	0.01	0.04
	Distractor Foveation Time	− 0.06	0.05	< 0.01	< 0.01	0.23	0.07
	Hand-Area Bias	− 0.02	0.04	< 0.01	< 0.01	0.65	0.10
Distractors Avoided	Gaze-Hand Distance	0.29	0.04	< 0.09	< **0.25**	< 10^–6^	0.65
	Target Foveation Time	− 0.11	0.04	< 0.01	< **0.04**	< 10^–3^	0.23
	Hand-Speed Bias	0.11	0.03	< 0.01	< **0.03**	< 10^–3^	0.51
	Extrafoveal Hits	0.11	0.03	< 0.01	< **0.03**	< 10^–3^	0.37
	Foveation Time Difference	− 0.09	0.03	< 0.01	< **0.02**	< 10^–3^	0.31
	Objects Foveated	0.09	0.04	< 0.01	< **0.02**	< 0.01	0.21
	Distractor Foveation Time	0.05	0.04	< 0.01	< 0.01	0.19	0.32
	Hand-Area Bias	0.02	0.03	< 0.01	< 0.01	0.54	0.25
	Mean Hand-Speed	0.01	0.04	< 0.01	< 0.01	0.92	0.14

**Fig. 8:**
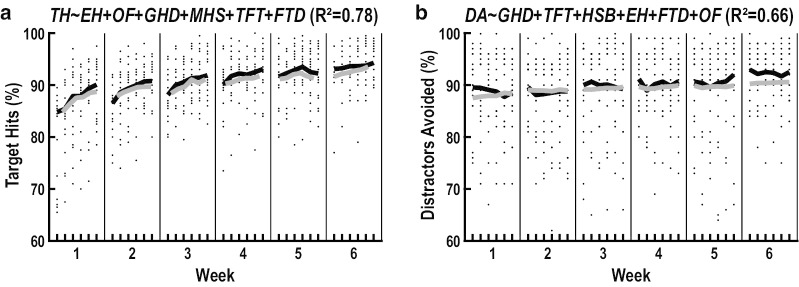
Multivariate predictions of task performance. Illustrations show the predicted improvements in Targets Hit (**a**) and Distractors Avoided (**b**) obtained from multiple regression. Each panel shows raw data values of all individual participants (small black dots), group means of the reweighted participant data (thick black lines), and model predictions (thick grey lines). Reweighted participant data were obtained by applying weights from the robust regression to the raw data values of all participants

We subsequently tested our second hypothesis by using multiple regression to analyze the extent to which refinements on the preceding predictor measures were independently predictive of improvements in Target Hits and Distractors Avoided (i.e., at least a small semipartial effect size, ${f}^{2}$≥0.02) (Table 4). Our multiple regression identified two measures of visual search (Extrafoveal Hits: $\beta$=0.54, ${f}^{2}$=0.61, $sp$<10^–6^; Objects Foveated: $\beta$=0.32, ${f}^{2}$=0.16, $sp$<10^–6^), one measure of eye-hand coordination (Gaze-Hand Distance: $\beta$=0.22, ${f}^{2}$=0.07, $sp$<10^–3^), and one measure of skilled limb movement (Mean Hand-Speed: $\beta$=0.14, ${f}^{2}$=0.03, $sp$=0.02) that were independently predictive of improvements in Target Hits (Fig. 8a). In contrast, our multiple regression only identified a single measure of eye-hand coordination (Gaze-Hand Distance: ($\beta$=0.24, ${f}^{2}$=0.04, $sp$=0.01) that was independently predictive of improvements on Distractors Avoided (Fig. 8b).

**Table 4:** Multiple regression between predictor and outcome measures

Outcome		*DF*	${R}^{2}$	${F}^{2}$	$P$	$P$_(residuals)_
Target Hits	Full Model	631	0.78	3.59	< 10^–6^	0.71
	*Predictors*	$\beta$	${sr}^{2}$	$s{f}^{2}$	$sp$	*Tolerance*
	Extrafoveal Hits	− 0.54	0.133	**0.61**	< 10^–6^	0.83
	Objects Foveated	− 0.32	0.034	**0.16**	< 10^–6^	0.88
	Gaze-Hand Distance	− 0.22	0.015	**0.07**	< 10^–3^	0.83
	Mean Hand-Speed	− 0.14	0.006	**0.03**	0.02	0.89
	Target Foveation Time	− 0.11	0.003	0.01	0.12	0.72
	Foveation Time Difference	− 0.07	0.002	0.01	0.08	0.79

Outcome	Predictors	*DF*	${R}^{2}$	${F}^{2}$	$P$	$P$_(residuals)_
Distractors Avoided	Full Model	631	0.66	1.90	< 10^–6^	0.48
	*Predictors*	$\beta$	${sr}^{2}$	$s{f}^{2}$	$sp$	*Tolerance*
	Gaze-Hand Distance	− 0.24	0.014	**0.04**	0.01	0.86
	Hand-Speed Bias	− 0.09	0.005	0.01	0.03	0.95
	Extrafoveal Hits	− 0.04	0.001	0.00	0.32	0.83
	Foveation Time Difference	− 0.04	0.001	0.00	0.40	0.90
	Target Foveation Time	− 0.01	0.000	0.00	0.94	0.74
	Objects Foveated	− 0.01	0.000	0.00	0.95	0.88

Bold indicates measures that exhibited meaningful relationships with Targets Hit or Distractors Avoided (*sf*^2^ ≥ 0.02).

Finally, our stepwise regression analyses confirmed the results obtained from our multiple regression analyses. Specifically, the final model for Targets Hit only included the same measures of visual search (Extrafoveal Hits, Objects Foveated), eye-hand coordination (Gaze-Hand Distance) and skilled limb movement (Mean Hand-Speed). Furthermore, the final model for Distractors Avoided only included Gaze-Hand Distance.

## Discussion

### Multiple processes independently predict motor learning

The results of this study provide indirect evidence that practice-related refinements involving multiple neural processes may contribute to motor learning. Notably, we observed that measures of skilled limb movement, visual search and eye-hand coordination underwent practice-related refinements (Hypothesis 1) that were independently predictive of improvements in task performance (Hypothesis 2). Importantly, in drawing this conclusion, we assume that the trial-by-trial and week-by-week refinements exhibited by measures of skilled limb movement, visual search and eye-hand coordination can be used to infer that practice produced refinements involving multiple neural processes. Furthermore, we assume that motor learning could be inferred from trial-by-trial and week-by-week improvements exhibited by measures of task performance.

Other studies have provided evidence that both sensory and motor processes contribute to motor learning [48], but these studies were not designed to investigate the extent to which these processes are independent predictors of motor learning. As result, we do not know the extent to which relationships with motor learning reflected independent or shared variance. In the current study, we addressed the issue of covariation by examining independent predictions of motor learning after removing all shared variance. This analysis showed that skilled limb movements, visual search and eye-hand coordination are independent predictors of motor learning, indicating that studies of motor learning should account for the various processes that may influence improvements in task performance.

### Skilled limb movements independently predict motor learning

Increases in Mean Hand-Speed were associated with increases in Targets Hit, indicating that participants learned to hit more targets by quickly moving their hands to different areas of the workspace. Although faster movements are more variable and less accurate [2, 9], any decreases in movement accuracy were not associated with increases in the proportion of hand movements that failed to make paddle-contact with targets. Alternatively, it is possible that optimization of intermuscular coordination may have allowed participants to move faster without incurring greater movement variability. In either case, increases in movement speed had a positive effect on task performance, thus our results are consistent with the principles of optimal feedback control [49, 50].

### Visual search independently predicts motor learning

Increases in Extrafoveal Hits and Objects Foveated were the strongest independent predictors of increases in Targets Hits. These findings indicate that refinements of visual search led to better task performance by optimizing how participants gathered information with foveal and extrafoveal vision. This is consistent with evidence that visual search is highly adaptive to different task demands and environments, such as environments in which task-relevant objects are more likely to appear at certain locations [51, 52].

The association between Extrafoveal Hits and Target Hits indicates that participants learned to use extrafoveal information to guide hand movements used to hit targets. This is consistent with a previous study of visual search, which found that practice led to improvements in using extrafoveal vision to search for objects with task-relevant features [53]. In addition, cortical areas known to process peripheral visual information exhibit greater involvement during motor tasks [54]. However, to our knowledge, our study is the first to show that refinements of extrafoveal visual processing are predictive of motor learning.

The association between Targets Foveated and Target Hits suggests that refinements used to maximize the number of objects that participants foveated with visual search led to improvements in hitting targets. The modest correlation between Objects Foveated and Target Foveation Time (*r* = − 0.31; Table 1) also indicates that, at least in part, decreases in the time spent foveating targets freed up time to foveate more objects. In contrast, studies of “quiet eye” have found that experts at motor tasks have longer foveations on task-relevant objects than novices [17, 19, 20]. Furthermore, training interventions designed to increase foveation durations have produced improvements in motor performance [22–25]. These divergent findings suggest that both increases and decreases in foveation times can benefit motor performance, depending on the task demands and environment. As a result, we predict that practice will lead to increases in target foveation times in tasks with high demands on accuracy and low demands on speed of visual processing, whereas practice will produce decreases in foveation times in tasks with low demands on accuracy and high demands on speed of visual processing.

### Eye-hand coordination independently predicts motor learning

Increases in Gaze-Hand Distance were associated with increases in Targets Hits, indicating that looking away from targets before hitting them led to improvements in task performance. Although this contrasts with studies showing rigid coupling between initiation of eye movements and completion of hand movements [43], other studies have found that this rigid coupling decreases with practice [33–36]. We believe that increases in Gaze-Hand Distance may reflect a transition from an early reliance on visual feedback for accurate execution of hand movements to a subsequent reliance on kinesthetic feedback for accurate execution of hand movements. This would have allowed visual search to gather task-relevant information with greater efficiency [33]. Specifically, looking away from targets before hitting them would have disrupted visual feedback used to accurately guide hand movements toward targets. However, it would have enabled earlier and longer foveations of objects, thereby facilitating more efficient decisions whether to hit or avoid objects by either executing or inhibiting skilled limb movements. Importantly, any negative effects resulting from disruption of visual feedback of hand movements could be offset by a greater reliance on kinesthetic feedback, which is known to improve during motor learning [55–57] and may directly contribute to motor learning [58–61].

### Distinct predictors of motor execution and inhibition

We found that motor execution (Targets Hit) and motor inhibition (Distractors Avoided) exhibited distinct patterns of improvements. Notably, Targets Hit showed trial-by-trial and week-by-week improvements, whereas Distractors Avoided displayed only week-by-week improvements. We also found that different processes were independently predictive of improvements in motor execution and inhibition. Refinements of skilled limb movements (Mean Hand-Speed), visual search (Objects Foveated, Extrafoveal Hits) and eye-hand coordination (Gaze-Hand Distance) were independently predictive of improvements in Targets Hit. In contrast, eye-hand coordination (Gaze-Hand Distance) was the only independent predictor of improvements in Distractors Avoided. Given that avoiding distractors mainly involved inhibition rather than execution of hand movements, it is not surprising that increases in Mean Hand-Speed were not predictive of increases in Distractors Avoided. In contrast, increased Gaze-Hand Distance would have facilitated both motor execution and inhibition by allowing participants more time to make decisions whether to initiate or inhibit movements. It is perhaps surprising that increases in Objects Foveated were not predictive of increases in Distractors Avoided. We would expect that more efficient visual search should lead to improvements in both motor execution and inhibition by allowing more objects to be processed with foveal vision. The lack of a relationship may reflect that participants exhibited smaller improvements on Distractors Avoided. However, if the proportion of targets and distractors was equal or reversed, we expect that our participants may have shown greater improvements on Distractors Avoided and we may have found a meaningful relationship.

### Limitations

By examining patterns of variability exhibited by measures related to multiple neural processes, we found that refinements of multiple processes were independently predictive of motor learning. However, our paradigm and analyses were not designed to make causal inferences. This requires measuring motor learning while experimentally manipulating one process and controlling for interactions with all other processes. For example, masking objects that are not located within foveal vision would neutralize the contributions of extrafoveal hits on motor learning. If this reduced motor learning without affecting refinements of other processes, it would show that refinements of extrafoveal processing are causally linked to motor learning.

Another limitation of the current study is that we did not examine practice-related refinements of proprioception. This is an important limitation because improvements in planning and executing skilled limb movements may involve refinements that alter the processing of proprioceptive feedback [37]. In agreement with this hypothesis, previous studies have demonstrated that motor learning is associated with modifications of rapid responses to proprioceptive feedback [62] and improvements in kinesthesia [54–56]. Although we do not know if refinements involving proprioceptive processing contribute to motor learning in the current study, we believe they may have facilitated increases in Gaze-Hand Distance by reducing reliance on visual feedback used to accurately execute skilled limb movements.

Task demands and environmental features are known to alter motor learning [63, 64]. However, we did not investigate how task demands and environmental features influence the extent to which different processes are predictive of motor learning. In the current paradigm, for example, we would expect that refinements of skilled limb movements would be a greater predictor of motor learning if the demands on skilled limb movements were increased by reducing the size of the paddles or by imposing mechanical perturbations on the hands.

Although our behavioral measures probed several neural processes involved in motor learning, we did not directly investigate the underlying neural mechanisms of motor learning. Numerous studies of motor learning have explored changes in brain regions and networks related to refinements of skilled limb movement [65–67]. Other studies have investigated the brain regions and networks associated with visual search during perceptual and cognitive tasks [68–72]. However, we are unaware of any studies that have examined the extent to which brain regions and networks that underlie multiple processes are associated with motor learning.

## Conclusions

Our findings indicate that motor learning may result from refinements of multiple behavioral features that are mediated by adaptations involving multiple neural processes. This knowledge may help advance post-stroke rehabilitation. Notably, most stroke survivors experience chronic difficulties performing daily motor tasks like cooking, walking, and driving [73–75], and many exhibit deficits in performing skilled limb movements [76–80], visual search [81–83], and eye-hand coordination [84, 85]. Our findings suggest that these deficits may independently alter the outcomes of rehabilitation interventions designed to target mechanisms of motor learning. Future studies that investigate the extent to which these deficits independently affect motor learning are needed to guide the development of novel neurorehabilitation interventions that can improve motor function and reduce chronic disability after stroke.

## Data Availability

All data used for the current study will be made available by the corresponding author on request by any qualified researcher.

## Abbreviations

OHA: Object hit and avoid
BIC: Bayesian information criterion
